# Just enough fruit: understanding feedback mechanisms during sexual reproductive development

**DOI:** 10.1093/jxb/erad048

**Published:** 2023-02-01

**Authors:** Avi Sadka, Catriona H Walker, Dor Haim, Tom Bennett

**Affiliations:** Department of Fruit Tree Sciences, Institute of Plant Sciences, ARO, The Volcani Institute, Rishon Le’Zion 7528809, Israel; School of Biology, Faculty of Biological Sciences, University of Leeds, Leeds LS2 9JT, UK; Department of Fruit Tree Sciences, Institute of Plant Sciences, ARO, The Volcani Institute, Rishon Le’Zion 7528809, Israel; The Robert H. Smith Faculty of Agriculture, Food and Environment, The Hebrew University of Jerusalem, Rehovot, 76100, Israel; School of Biology, Faculty of Biological Sciences, University of Leeds, Leeds LS2 9JT, UK; University College Dublin, Ireland

**Keywords:** Correlative inhibition, dominance, flowering, fruit, plant reproductive development

## Abstract

The fruit and seed produced by a small number of crop plants provide the majority of food eaten across the world. Given the growing global population, there is a pressing need to increase yields of these crops without using more land or more chemical inputs. Many of these crops display prominent ‘fruit–flowering feedbacks’, in which fruit produced early in sexual reproductive development can inhibit the production of further fruit by a range of mechanisms. Understanding and overcoming these feedbacks thus presents a plausible route to increasing crop yields ‘for free’. In this review, we define three key types of fruit–flowering feedback, and examine how frequent they are and their effects on reproduction in a wide range of both wild and cultivated species. We then assess how these phenomenologically distinct phenomena might arise from conserved phytohormonal signalling events, particularly the export of auxin from growing organs. Finally, we offer some thoughts on the evolutionary basis for these self-limiting sexual reproductive patterns, and whether they are also present in the cereal crops that fundamentally underpin global diets.

## Teaser

A review examining how fruit produced early during reproduction can inhibit the formation of new fruit later in development, and why this may be advantageous for plants.

## Introduction

There are some ideas that every gardener holds to be true. Ornamental flowers should be ‘dead-headed’ to keep them flowering. Pea pods should be promptly picked to promote production of further fruit. The ‘June drop’ will see some proportion of fruit falling from orchard trees, long before ripening. But conversely, if a tree is allowed to retain too much fruit, the tree might take a break from fruit production in the next year. The typical gardener is probably happy to ignore exactly how and why these effects occur. And yet, in formulating these guidelines, growers nevertheless seem to appreciate something fundamental about the coordination of plant growth and development—that different organs, at different times, can exert a profound inhibitory effect on the production or maintenance of other organs. Indeed, these inter-organ feedbacks are a critical component of the way in which plants organize their architecture so as to grow optimally in relation to resource availability in the environment, providing a developmental framework that is robust and yet highly flexible.

The aim of this review is to explore these feedback-type mechanisms specifically in the context of reproductive development—to explore feedback that occurs between fruit and flowers, and how these allow plants to optimize their reproductive success in an uncertain environment. We will start by trying to define the types of feedback that occur, before considering the mechanisms that underlie these relationships, exploring their adaptive benefits, and assessing the prospects for using this information to improve crop yields.

## Fruit-driven feedback

**Fig. 1.**
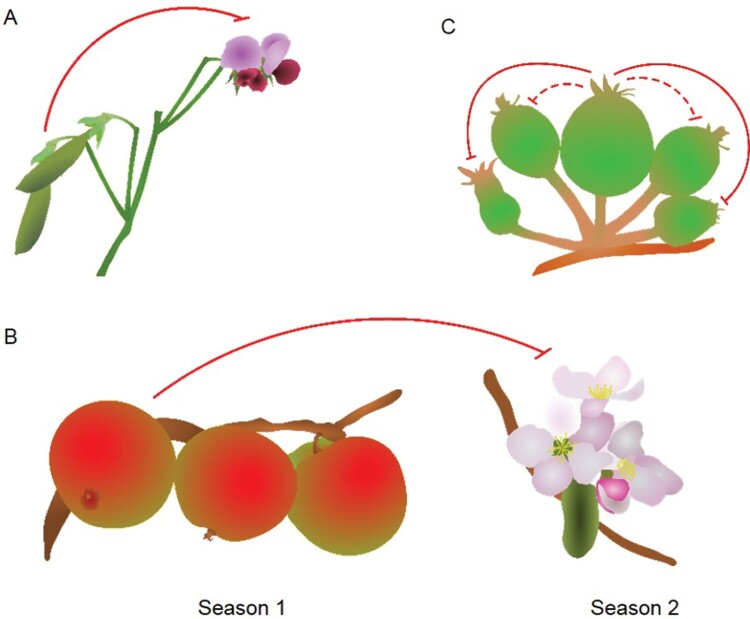
The three key types of fruit-driven feedback across species. (A) In pea, developing fruit are capable of inhibiting the formation of later-produced flowers, in same-phase feedback. (B) Apple displays next-phase feedback, where the fruit produced one season can inhibit the production of flowers in the subsequent season, resulting in ‘alternate bearing’. (C) In apple fruit–fruit feedback, the ‘dominant’, ‘king’ fruit inhibits the development of other local fruit, sometimes leading to their abscission from the tree.

The production of fruit can have several potent effects on the reproductive effort of a mother plant. Since parthenocarpic (unfertilized) fruit can in many cases cause the same or similar effects as fertilized fruit, it is clear that fruit can exert significant feedback in their own right. However, fertilized fruit often have a stronger effect that parthenocarpic fruit (reviewed in Bangerth, 1989), and certainly in non-parthenocarpic varieties the presence of seed is essential for feedback to occur. In this review, we will use ‘fruit’ as a shorthand for ‘fertilized fruit’ unless otherwise stated, and therefore the feedback effects we describe are typically the effects caused by the combined fruit and seed. Fruit-driven effects can be broadly divided into three key types: feedback on flowering in the same reproductive phase (same-phase feedback), feedback on the subsequent reproductive phase (next-phase feedback), and feedback on the growth or retention of other fruit (fruit–fruit feedback) (Fig. 1A)

## Same-phase feedback

The routines of dead-heading and pod-picking illustrate the growers’ intuition that allowing plants to set fruit will *somehow* limit the ongoing reproductive effort of the plant. However, while the effects of fruit on fruit-set and subsequent reproductive phases are well established in many species in the scientific literature, stimulation of *ongoing* flowering by fruit removal is, to the best of our knowledge, much less well-described. Indeed, the notion that flower- or fruit-picking promotes ongoing flowering seems largely anecdotal, based on experience of growing peas and beans in particular. Nevertheless, work in the *Brassicaceae*, and in particular in the model species Arabidopsis, suggests that these effects do occur, although they may be relatively subtle.

Before examining the evidence, it is perhaps useful to define the ways in which plants can continue their reproductive effort. To produce more flowers, plants can initiate more inflorescences (of both lower and higher orders); they can initiate more floral primordia on existing inflorescences; or they can fully develop a larger number of the floral primordia (in essence, they can ‘open more flowers’) (Fig. 1) (González-Suárez *et al.*, 2020). Amongst different species, the structural differences in the arrangement and type of inflorescence may restrict these possibilities. For instance, in species that produce small inflorescences with a relatively fixed number of flowers, the principal option to sustain flowering will be to initiate new inflorescences. Therefore in peas, for example, where two flowers are typically initiated and opened on each inflorescence, only production of new inflorescences can sustain flowering.

The simplest demonstration that fruit-set affects ongoing flowering comes from analysis of male-sterile mutants in Arabidopsis, which do not self-pollinate, and as such do not set seed unless manually cross-pollinated. These mutants, for instance *male sterile1* (*ms1*), flower for a considerably longer period than wild-type, and in the process, initiate more inflorescences than wild-type (Hensel *et al.*, 1994). Those inflorescences in turn produce more total floral primordia than wild-type, and open a higher proportion (~100%) of those primordia (Hensel *et al.*, 1994). The plants, in response to the lack of fertile fruit, seem to ‘throw everything’ into their reproductive effort. The complete removal of fruit from wild-type plants promotes a similar flowering response, as does the lack of viable seed in gametophytic lethal mutants (which are fertile, but only have ~25% seed set) (Hensel *et al.*, 1994).

**Fig. 2.**
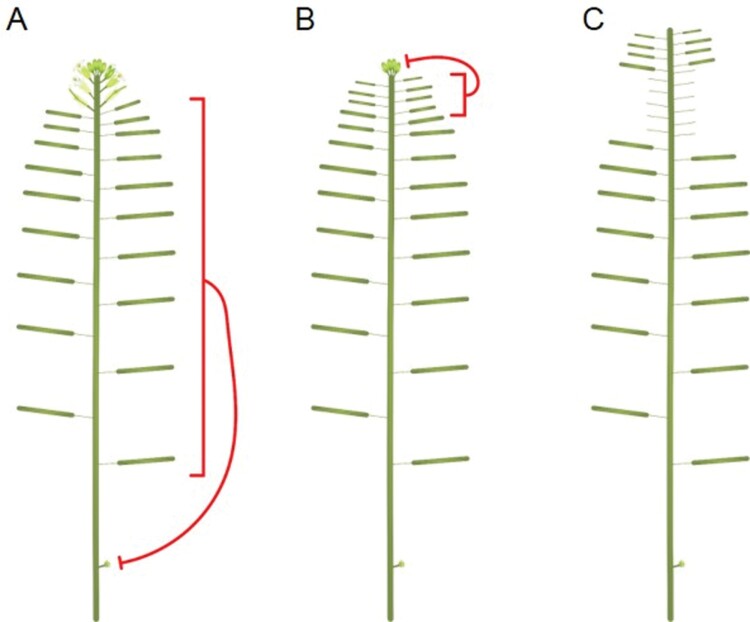
Arabidopsis fruit inhibition regulates future flowering. (A) The presence of seed-containing fruit on an inflorescence inhibits the development of buds, which would form a higher-order inflorescence if allowed to develop. (B) Seed-containing fruit proximal to the bud cluster act to inhibit flower development, bringing about inflorescence arrest in Arabidopsis. (C) Removal of fruit proximal to the inflorescence meristem around the time of inflorescence arrest results in the extended opening of floral primordia present within the bud cluster, which would not otherwise develop.

Further investigation has confirmed and delineated the effects of fruit on Arabidopsis flowering. Fertile fruit located on a given inflorescence exert an inhibitory effect on the outgrowth of subtending, high-order inflorescences on the same branch (Fig. 2A) (Walker *et al.*, 2021). This is part of a more generalized ‘infloretic dominance’, in which the major inflorescences inhibit the activation of their own higher-order, minor inflorescences, and to which the major inflorescence meristem (IM) also contributes (Walker *et al.*, 2021). The effect of each fruit is very small, such that at the beginning of inflorescence lifetime, the effect of fruit is negligible. However, by the time IM arrests, sufficient fruit have usually been formed that their collective effect is sufficient to maintain the inhibition of minor inflorescence activation (Walker *et al.*, 2021). Fruit appear to exert a minimal influence on the activity of the IM on the same inflorescence, such that removal of fruit produced while the IM is active has no discernible effect on the number of floral primordia initiated by the IM (Wang *et al.*, 2020; Walker *et al.*, 2022) (Fig. 2B). Where Arabidopsis fruit do exert a more noticeable effect is on the flower maturation/flower opening process. Arabidopsis inflorescences typically arrest with a cluster of ~15 unopened buds (Hensel *et al.*, 1994), but removal of later-formed fruit is sufficient to promote the opening of almost all of the remaining flowers on the same inflorescence (Ware *et al.*, 2020; Walker *et al.*, 2022). This process does not involve renewed IM activity, and seems to reflect the very localized inhibitory effect that fruit have on the development of nearby floral primordia (Walker *et al.*, 2022) (Fig. 2C).

Overall, the data from Arabidopsis support the idea that fruit can inhibit the progression of the ongoing flowering, but the effects of Arabidopsis fruit are rather mild. This might specifically reflect the small size of Arabidopsis fruit, and the short duration of its floral period, or might generally reflect the relevant unimportance of same-phase feedback. Clearly, more experiments are needed across a range of species with different reproductive shoot architectures to establish how generalizable these data are. Pea seems an obvious place to start, since it has long been implicated in these phenomena, and is a highly tractable experimental organism.

## Next-phase feedback

Next-phase feedback most obviously occurs in perennial plants in which there is an annual sequence of flowering, with some temporal separation between production of fruit in one year and flowering of the following year, as opposed to repeated waves of flowering during a single year. Next-phase feedback has been particularly studied in commercial fruit trees, in which it can have a major effect on crop yield. In general, based on their annual cycles, two major types of fruit trees can be defined: deciduous and evergreen. While both undergo a synchronous ‘blossoming’ (flower opening) in spring, the induction of flowering differs significantly between them. Flowering induction of deciduous trees usually occurs 80–90 d following anthesis of the previous generation of flowers, most likely due to endogenous developmental cues (Goeckeritz and Hollender, 2021). Inflorescences are initiated, and flower buds form and differentiate before entering paradormancy and spending winter as arrested floral buds. Conversely, floral induction of evergreen subtropical fruit trees occurs during the winter, where the accumulation of sufficient number of cold hours plays a major role in the conversion of vegetative meristems into IMs (Wilkie *et al.*, 2008). Flowering induction in tropical fruit trees also results from endogenous cues, but is not always synchronized to a certain season. In some fruit trees there is clear phase separation, and fruit are harvested prior to the subsequent flowering induction period; for instance, olive and early citrus cultivars. However, in many trees, there is no clear phase separation, and fruit are still present on the tree during or following the subsequent flowering induction; for instance, most citrus cultivars, avocado, and mango. There are even extreme cases where the previous season’s fruit is present during flowering, fruit set and early stages of fruit development, particularly Valencia orange. In all these scenarios, the fruit can exert an inhibitory effect on the subsequent flowering period. Most obviously, these inhibitory effects occur at the level of floral induction, with fruit dampening down the number of inflorescences produced the following season, even if the fruit are no longer present, implying the existence of a remarkable ‘fruit memory’ (Goldschmidt and Sadka, 2021). However, fruit presence can also impact on subsequent stages of flowering, flower development, and even bud break (Verreynne and Lovatt, 2009).

**Fig. 3.**
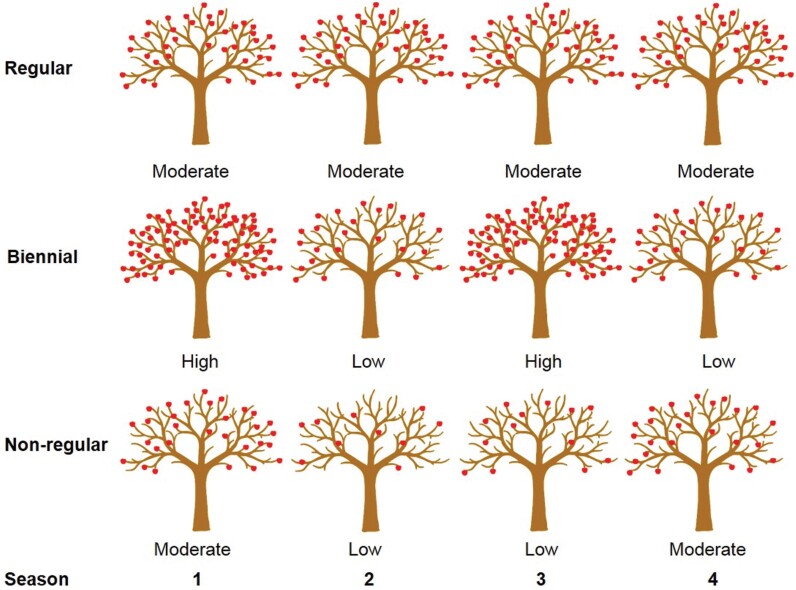
Bearing patterns of fruit trees over multiple seasons. Biennial bearing results in alternate years of high and low yield, unlike regular bearing patterns, where yields are more stable and consistent. Non-regular bearing trees tend to have non-regular, less predictable yield from season to season.

The effect of next-phase fruit feedback is clearly seen in cultivated fruit trees that have an ‘alternate bearing’ (AB) habit (reviewed in Goldschmidt and Sadka, 2021). This refers to the common case in fruit trees when fruit yield of one year inhibits flowering the following year. Moderate yield fluctuations are normal in every cultivar of any fruit species, and occur in response to environmental cues. However, what defines AB is the extreme fluctuation in yield, with heavy fruit load one year (on-crop) followed by very low yield (off-crop) the following year (Fig. 3). Nevertheless, unfavourable climatic conditions, especially during flowering and fruit set of the on-crop year, may cause flowers or fruitlets to drop, and cause a consecutive on-crop year. Additionally, fruit thinning or complete removal (de-fruiting) before the subsequent floral induction period also induces a consecutive on-crop year (back flowering), demonstrating that AB is primarily driven by feedback from fruit. Even in perennial plants without the AB habit, it is likely that fruit-set exerts some feedback on the subsequent reproductive phase, but the phenomenon is most easily visible (and therefore studied) in AB cultivars. De-fruiting or thinning during the on-crop year needs to be performed a significant period before the onset of floral induction, otherwise the plant will ‘remember’ the presence of the fruit even in their absence. How early the de-fruiting needs to be performed is species-dependent. For instance, under the east Mediterranean climate, de-fruiting before October is effective in inducing back flowering in avocado (Ziv *et al.*, 2014). In most citrus cultivars, fruit removal is effective if performed before November (Muñoz-Fambuena *et al.*, 2011). Therefore, it could be concluded that fruit memory in citrus and avocado becomes fixed until about 1 to 2 months before the onset of floral induction. In olive, fruit memory becomes fixed earlier, and fruit removal is effective only if performed before mid- or, at most, the end of August (Dag *et al.*, 2010; Haberman *et al.*, 2017). The mechanisms by which fruit memory affects flowering are still enigmatic. In fact, the mechanistic question can be divided into two: first, how the meristem or the bud senses fruit presence, and second, how this sensing mechanism is translated into a decision-making process: flowering promotion or inhibition. These two topics are discussed below (section ‘Auxin canalization—the origin of dominance?’).

The feedback between fruit and flowering plays an important role in allowing perennial plants to balance their growth between vegetative and reproductive development. In most perennials, inflorescences are initiated as specialized lateral branches on vegetative shoots, and therefore annual vegetative growth is a prerequisite for ongoing productive capacity. In many fruit trees, flowers occur on 1-year-old shoots, emphasizing the need for ongoing vegetative capacity (Bangerth, 2009). However, over-flowering may impair vegetative growth, and vice versa. Regular bearing plants seem to use two mechanisms to control a proper balance between vegetation and productivity. The first, fruitlet thinning (see below) provides an important mechanism to control the final number of fruit in each season, which might normally exceed available resources anyway (for instance, ‘Wilking’ mandarin lacks this ability, and if no chemical thinning is applied, the tree may die while carrying many fruit) (Goldschmidt and Sadka, 2021). The second mechanism could be defined as proportional allocation of resources between vegetative growth and reproduction, which occurs under the influence of the remaining fruit. In this context, it can by hypothesized that AB represents a highly simplified strategy to avoid the problem of resource allocation, in which no proportional allocation is required, as the case in regular bearer tree. Resources are allocated one year for vegetative growth (off-crop year) and for productivity the following year (on-crop year). Testing this hypothesis provides a challenge, as it requires full understanding of the meaning of ‘economic’ cost of resource allocation between vegetative and reproductive growths.

From the growers’ perspective, non-regular bearing presents an economic problem, but in nature this might be the norm. Productivity of many forest trees follows a ‘masting’ phenomenon, where extreme but synchronized fluctuations in yield are detected (Kelly and Sork, 2002). Productivity varies between species, with 2–3-year cycles in chestnut, hazelnut, and elm and up to 10–15 years in beeches. The productivity of wild relatives of fruit trees has been reported only in a few cases (reviewed in Goldschmidt, 2013b, 2018). Yield of wild apple (*Malus sieversii)* varies greatly from tree to tree, and seems to be affected by many environmental parameters, but when trees of this species were planted in an experimental farm, their multi-annual productivity was quite stable, and they did not show a biennial bearing pattern (Goldschmidt, 2013a). Conversely, Polish wild pear (*Pyrus pyraster*) and wild olive showed clear biennial and non-regular bearing patterns (Goldschmidt, 2013a). The pecan nut (*Carya illinoiensis*) and macadamia (*Macadamia tetraphylla* and *M. integrifolia*) provide examples from recently domesticated fruit trees. In the wild, pecan displays masting and consistent with this, cultivated trees show strong alternate bearing behaviour. Conversely, in the wild, macadamia produces very low and non-regular yield, but when cultivated, the trees show relatively high and stable yield (Goldschmidt, 2013a). These cases suggest that alternate or non-regular bearing is common in the wild, and likely results from the interaction between endogenous and exogenous cues. However, in the wild, productivity varies from tree to tree due to interactions between local conditions and endogenous cues. Only under cultivation, when selected uniform genotypes are grown under homogeneous practices, is the true nature of the production strategy revealed—regular, biennial, or non-regular (Goldschmidt and Sadka, 2021).

Stable and sufficient multi-annual yield is an obvious prerequisite for cultivation of any fruit tree. Therefore, domestication of fruit trees selected against non-regular bearing of any type (Goldschmidt, 2013a, 2018). For instance, the domestication of macadamia, an extreme case of a non-productive wild tree, was based on multiple selections of lines with increased yield. However, selecting against biennial bearing has proved more difficult, with the trait present in many modern cultivars. Molecular markers for regular bearing could therefore be useful in breeding programmes. Indeed, genetic and bioinformatics analyses of segregating populations between a strongly biennial bearing apple cultivar and regular bearer cultivar have identified quantitative trait loci (QTLs) that could explain phenotypic variability associated with biennial bearing, leading to the development of tools to access bearing behaviour, at least during the first year of tree maturity (Guitton *et al.*, 2012; Durand *et al.*, 2013, 2017). However, so far, to the best of our knowledge, these tools are not practically used. Moreover, to the best of our knowledge, these are the only reported attempt to generate marker-assisted breeding for stable yield traits, and no markers have been reported in other species. Indeed, in many breeding and selection programmes, non-regular bearer trees are not discarded if they meet other desired criteria. It should also be considered that for most species and cultivars, efficient horticultural practices to improve productivity have been such that in many cases, the ‘domestication force’ against non-regular bearing is not based on genetic selection, but on horticultural practices.

## Fruit feedback on fruit growth and retention

The last major type of fruit-driven feedback is the effect of older fruit on the growth and retention of younger fruit (fruit–fruit feedback, FFF). FFF could theoretically be exerted at three scales: within inflorescences, between inflorescences on the same branch, and between inflorescences on different branches. In annuals, FFF can sometimes be observed at all scales, but in fruit trees FFF is mostly noticed and studied at the intra-inflorescence level and between inflorescences on the same branch. FFF has an important economic impact as it affects the overall number of fruit and it alters the ratio between small and large fruit, thus affecting yield (Cockshull and Ho, 1995).

Cucurbits provide some of the most striking examples of FFF, with a single fruit often inhibiting the development of all subsequently initiated fruit, in both seeded and parthenocarpic varieties (Baniel *et al.*, 2008; Shnaider *et al.*, 2018). Inhibited fruit show a range of fates depending on their position in the hierarchy; older fruit show reduced or stalled growth, but younger fruit actively senesce (Shnaider *et al.*, 2018). Growth of inhibited fruit can, however, be restored by removal of the ‘dominant’ first fruit.

**Fig. 4.**
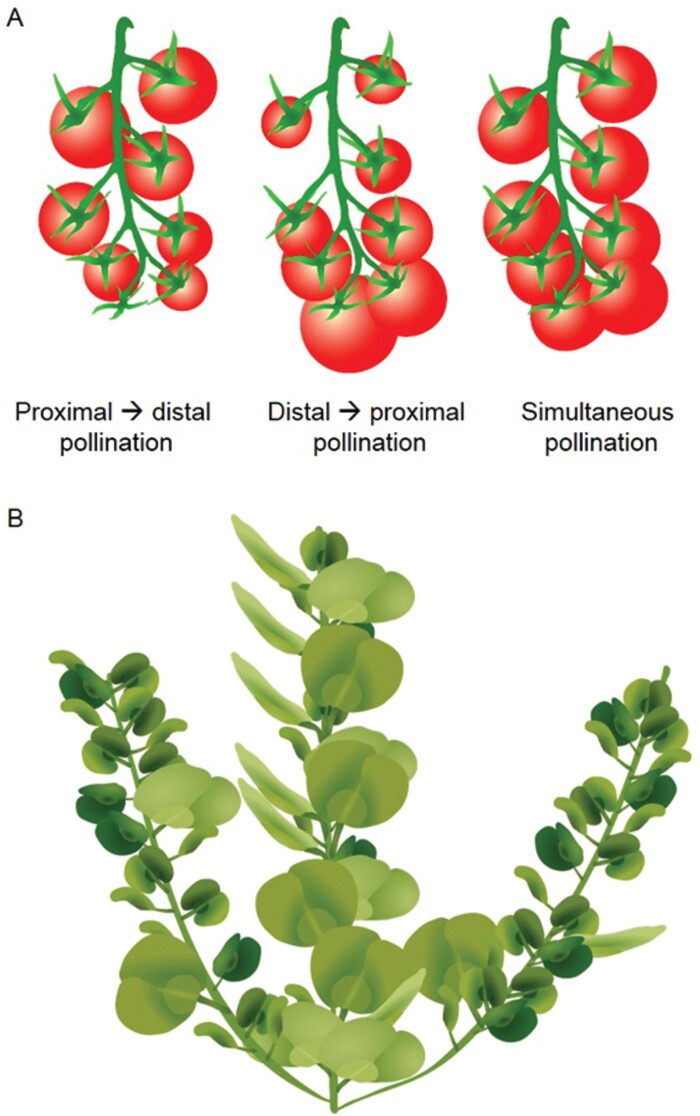
Fruit development regulates subsequent fruiting patterns. (A) Fruit size in tomato trusses is often determined by timing of pollination, with the earliest-pollinated flowers producing the largest fruit, regardless of position on the truss. Conversely, if all flowers are pollinated simultaneously, they all develop into fruit of similar mass. (B) *Aethionema arabicum* displays two distinct fruit morphs, with the primary inflorescence containing the majority of the large-morph fruit. The higher-order inflorescences typically contain predominantly the small-morph fruit. Removal of fruit or inflorescences can alter the distribution of these morphs across the plant, highlighting the dominance relationships between developing fruit (Lenser *et al.*, 2018).

In tomato inflorescences of indeterminate varieties, proximal fruit that are set earlier display a higher rate of assimilate import and have a larger number of cells than distal ones (Bangerth and Ho, 1984; Bohner and Bangerth, 1988). However, when the pollination sequence is manipulated, distal fruit appear earlier and become larger than proximal ones, and if all fruit are pollinated simultaneously, the fruit are the same size (Fig. 4A) (Bangerth and Ho, 1984). Within the inflorescence, fruit set failure is considerably higher in distal positions as compared with proximal ones (Bertin, 1995). Within the same truss, fruit number is also reduced due to abortion in later inflorescences as compared with earlier ones, unless thinning is performed. FFF between trusses is documented in tomato, but to a lesser extent than intra-inflorescence and between inflorescences; some reduction in fruit dry matter and growth rate is detected in later trusses than early ones (Bertin, 1995). While the above early works provide ‘physiological’ evidence for FFF, the accumulation of genetic and transgenic data provide further support. Numerous genes and QTLs are involved in tomato yield and individual fruit weight (reviewed in Ariizumi *et al.*, 2013). For instance, Fruit Weight 2.2 (FW2.2) is a transcription factor that inhibits cell division (Beauchet *et al.*, 2021). Due to sequence variation in its promoter region, it is expressed at higher levels in wild tomatoes than in cultivated lines, causing a small fruit phenotype. Generation of isogenic lines with the two promoter variants allowed assessment of their effect in a reference background (Nesbitt and Tanksley, 2001; Guo and Simmons, 2011). Fruit set is higher in the small fruit genotype, while flower abortion is lower if the number of flowers per inflorescence is greater than six. Interestingly, fruit thinning did not increase fruit weight and size showing the primary effect if FW2.2 is upon individual fruit size, and suggesting that effects on fruit number therefore result from FFF. Altering sink–source ratio was also performed by anti-sense manipulation of sucrose synthase, which along with cell wall invertase, plays an important role in generating sink strength of the fruit by controlling sugar import. The manipulation resulted in reduced fruit set, especially in second and third waves of flowering, and overall reduced number of fruit per plant and per truss (D’Aoust *et al.*, 1999). Regardless of the lower competition, fruit growth rate was also reduced, demonstrating that primary fruit feedback inhibition on later fruit does not always follow a simple inverse relationship between fruit number and size, especially when sink strength of the fruit and sink–source relationships are altered.

The *Brassicaceae* represent an interesting case for studying FFF, since some genera clearly display the phenomena, while others do not. For instance, *Aethionema* species produce two distinct fruit morphs as part of a bet-hedging strategy; larger fruit that immediately dehisce, for seed germination under currently favourable conditions, and smaller fruit that are indehiscent, with seeds for deposition in the long-term seed-bank (Lenser *et al.*, 2016, 2018). The larger fruit morph occurs mostly on main shoot (Fig. 4B), and the proportion of larger fruit could be further induced by side branch removal. Moreover, removal of the larger fruit from the main shoot induced the proportion of larger fruit on the side branches, indicating that indehiscent fruit arise through inhibition by dehiscent ones (Lenser *et al.*, 2018). Thus, *Aethionema* represents an interesting example where ‘inhibited fruit’ are repurposed as a secondary reproductive strategy. In *Brassica* species, FFF clearly exists, with many flowers opened later in the reproductive phase failing to set viable fruit under the influence of earlier fruit (Tayo and Morgan, 1975; Bangerth, 1989; Walker *et al.*, 2021). However, in Arabidopsis there is no discernible effect of early fruit on the growth or development of later-initiating fruit (Walker *et al.*, 2021).

In fruit trees, FFF within inflorescences is ubiquitous, and is associated with fruitlet self-thinning, which provides a natural mechanism aimed at regulating yield (Pawar and Rana, 2019). Fruit position within the inflorescence greatly determines its survival, as clearly demonstrated in apple, where about 70% of the fruitlets abscise. Fruitlets at the apical (‘king’) position have ~70% likelihood of surviving and developing into mature fruit, while those in lateral positions within the cluster have 70% likelihood to abscise (Maguylo *et al.*, 2014; Jakopic *et al.*, 2015). Moreover, mature fruit with immediate proximity to the king were smaller relative to both the king and those at lower positions, showing FFF is stronger with proximity to the king. Pedicels of fruit with lowest probability of abscising displayed extra vascular bundles, suggesting they play a role in dominance acquisition (Celton *et al.*, 2014). Inter-inflorescence feedback control is also demonstrated in fruit trees. The overall inverse relationships between the number of fruit and their growth rate, which is also a ubiquitous phenomenon, might be associated with fruit–fruit feedback control, regardless of relative position. In fig, for instance, proximal inflorescences develop into fruit and ripen earlier than distal ones; removal of the latter accelerates the development of the proximal inflorescences (Flaishman, 2022). Feedback control may also be exerted in species that flower and set fruit a few times a year. For instance, lemon trees usually bear large, harvestable fruit, along with small fruit originated from later flowering waves. Although the relationships between these two types of fruit have not been formally investigated, it is well acknowledged by growers that as soon as the large fruit are harvested, growth rate of the small ones is accelerated.

## Source–sink versus dominance relationships

A wealth of evidence indicates that fruit can exert strong feedback on flowering and fruit development, but how does this occur? In the next sections, we will focus on potential explanations for these effects. Fruit-driven feedback belongs to a class of phenomena often referred to as ‘correlative inhibition’, in which the growth of one organ (or class of organs) inhibits the growth of other organs (Bangerth, 1989). Probably the best-known class of correlative inhibition is ‘apical dominance’ in which actively growing shoot branches repress the activation of other shoot branches (Thimann and Skoog, 1933; reviewed in Domagalska and Leyser, 2011). There is a long running debate in the literature regarding the extent to which correlative inhibition is either driven by source–sink relationships, or by active ‘dominance’ mechanisms (Bangerth, 1989). The simplest and most intuitive model is the source–sink model, in which correlative inhibition arises because organs act as ‘sink’ organs for assimilates, of which there is a limited supply from ‘source’ organs. On the other hand, dominance mechanisms imply that organs actively inhibit the growth of other organs by signalling processes (Bangerth, 1989). In many ways, this debate reflects a broader debate on the nature of plants: are they passive in their environment, mutely reacting to changes in resource availability in a mechanical way? Or are they active in their environment, using information to proactively plan their growth to both current and future resource availability?

Like many such arguments, the source–sink/dominance debate very likely represents a false dichotomy. There is no reason to doubt that the availability of assimilates within the plant will influence and limit the growth of organs. But equally, to suggest that the only way plants regulate their growth is by waiting to run out of assimilates is to do a disservice to these incredibly complex organisms. There is now a large amount of evidence that plants use long-distance hormonal signalling, among a range of other mechanisms, to coordinate their growth, and there is no reason to persist which such a simplistic model. Indeed, modern formulations of source–sink models for plant growth fully integrate active hormonal signalling (Yu *et al.*, 2015; Chang *et al.*, 2017), and a reasonable argument can be made that one of the major functions of hormonal signalling is to organize the appropriate distribution of the limited supply of assimilates (Perez-Alfocea *et al.*, 2010; Shabala *et al.*, 2016). We take the attitude that, in the context of fruit-driven feedback, both passive source–sink relationships and active dominance mechanisms are likely to play a key role, to different extents in different organs. We will not examine source–sink models further, but will focus on the more active regulatory mechanisms of fruit-feedback.

## Auxin canalization—the origin of dominance?

In his seminal review and hypothesis paper on the subject of correlative inhibition, Bangerth (1989) proposed a simple mechanism by which an earlier-initiating organ can actively inhibit the growth of a later-initiating organ, regardless of their relative positions on the plant. He suggested that this ‘primigenic dominance’ arose by ‘auxin transport autoinhibition’ in which a strong auxin flux generated by the dominant organ inhibits auxin flow from a subordinate organ, increasing the hormone level in that organ, and inhibiting its growth (Bangerth, 1989). Thus, in Bangerth’s view, which he supported with a range of experimental observations, the relative ability of an organ to export auxin was key to determining both its own growth and its ability to feed back on the growth of other organs.

Research into the regulation of shoot branching, including the classic apical dominance phenomenon, has subsequently provided strong support for the general concepts proposed by Bangerth. Firstly, the ability of an individual shoot branch to grow is determined, at least in part, by its ability to export auxin (Morris, 1977; Crawford *et al.*, 2010; Balla *et al.*, 2011, 2016), and secondly, the ability of branches to inhibit other branches, and thus the total shoot branching level, is strongly dependent on the auxin transport environment in the shoot system (Bennett *et al.*, 2006, 2016; Crawford *et al.*, 2010; Shinohara *et al.*, 2013; Van Rongen *et al.*, 2019). Developing the auxin transport autoinhibition model further, these auxin-driven effects are usually now proposed to arise from auxin transport canalization, building on the original auxin canalization models for vascular patterning (Sachs, 1969, 1981; Bennett *et al.*, 2014). It is proposed that, in order to export auxin and therefore to grow, branches need to form or maintain a canalized auxin transport link to the main stem, and that their ability to do this will depend on the auxin source strength of the branch and the auxin sink strength of the main stem (Prusinkiewicz *et al.*, 2009). By extension, the ability of branches to inhibit other branches occurs because their auxin export weakens the auxin sink strength of the shared stem, limiting the ability of other branches to form canalized links (Prusinkiewicz *et al.*, 2009). Mathematical modelling demonstrates the plausibility of this (Prusinkiewicz *et al.*, 2009), and it is consistent with a wide range of experimental data, although it must be noted that it is far from universally accepted (reviewed in Waters *et al.*, 2017; Walker and Bennett, 2018).

Nevertheless, the canalization model for apical dominance provides a clear working model for fruit-driven feedback, which can be referred to as ‘carpic dominance’ in this context (Walker and Bennett, 2018). Indeed, carpic dominance was the context in which Bangerth originally framed his auxin transport autoinhibition model for primigenic dominance. There was sufficient evidence to suggest auxin as a causative agent when Bangerth proposed his ideas, but recent work has provided strong additional evidence that auxin transport plays a key role in carpic dominance. For instance, in the context of fruit load or alternate bearing, transcriptomic analysis of on- and off-crop buds in citrus identified induction of auxin polar transport genes in off-crop buds and in the buds of de-fruited on-crop trees soon after de-fruiting (Shalom *et al.*, 2012; Haim *et al.*, 2021). In association with this, indole-3-acetic acid (IAA) levels were higher in on-crop buds than off-crop buds, and this was reduced following de-fruiting (Shalom *et al.*, 2012; Haim *et al.*, 2021). The use of radiolabelled IAA in both citrus and olive has confirmed that auxin flux in the stem is higher when fruit is present and is reduced following de-fruiting (Haim *et al.*, 2021). Moreover, the auxin flux direction originated from the fruit, regardless of its position relative to the bud, and IAA removal from the bud was accelerated by fruit removal, providing further support for a canalization-based mechanism. In Arabidopsis, fruit have been shown to export significant quantities of auxin into the stem (Ware *et al.*, 2020), which progressively weakens auxin transport and increases auxin concentration in the upper inflorescence stem (Goetz *et al.*, 2021). These effects are necessary and sufficient to drive the fruit-induced arrest of the inflorescence (Ware *et al.*, 2020; Goetz *et al.*, 2021). Consistent with this, the inhibitory effect of early fruit on the development of later fruit in *Aethionema* species can be mimicked by auxin application, implying that auxin might drive this fruit–fruit feedback (Lenser *et al.*, 2018).

**Fig. 5.**
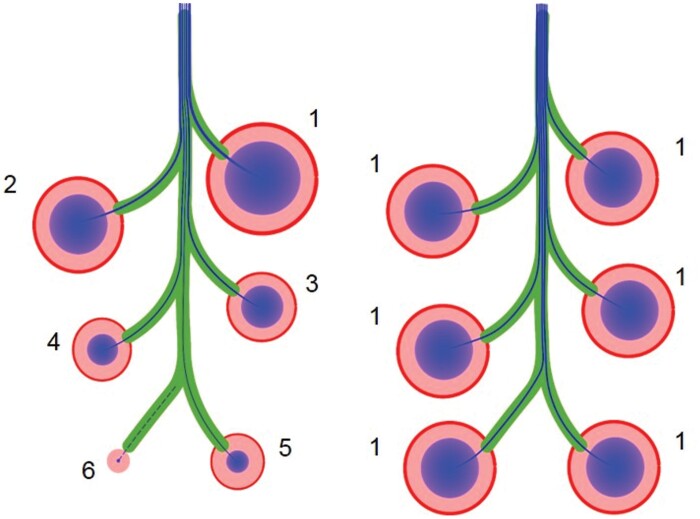
Model for auxin canalization in fruit development. Fruit are numbered in order of their development: sequentially (1–6) or simultaneously (1). Sequential development allows the earlier-developing fruit to export high levels of auxin into the stem, decreasing its sink strength. Subsequent fruit are therefore smaller, as they are unable to export high levels of auxin into the stem due to reaching ‘saturation’. Some fruit may be entirely inhibited from developing if they are unable to canalize to the polar auxin transport stream. Conversely, fruit developing simultaneously are all able to establish links to the polar auxin transport stream, resulting in more even auxin export from the fruit, and uniform fruit size.

However, as with apical dominance, the extent to which auxin export from fruit is the primary explanation for feedback on flowering and fruit development is still open. Considering the limited research tools in fruit trees, especially lack of genetic tools, the establishment of such direct relationships is not trivial. Application of auxin may alter flowering intensity and other flowering traits, such as the duration of bud break and the number of flowers in the inflorescence (Sadka *et al.*, 2022; Martinez-Fuentes *et al.*, 2022). Overall, the effects of auxin treatment are quite mild, probably due to the efficient ability of the plant to remove excess auxin (Eklöf *et al.*, 2000). While establishing more direct relationships between flowering control and auxin is challenging, the canalization model at least provides a testable framework on which to advance studies in this area (Fig. 5).

While auxin canalization provides the most reasonable scenario for dominance, the question can be asked whether the seed, fruit or both is the source of dominance. Auxin production within the fertilized ovule, and specifically in the endosperm, plays a major role in seed and fruit set (Figueiredo *et al.*, 2015; Guo *et al.*, 2022). Auxin accumulation is detected in fruit tissues during various developmental stages (Nishio *et al.*, 2010; Pattison and Catalá, 2012; McAtee *et al.*, 2013; Feng *et al.*, 2019). In early works, it was modelled that the source of dominance is within the seed (reviewed in Monselise and Goldschmidt, 1982). Apple displays negative correlation between the number of seeds per spur and percentage of flowering spurs (Dennis and Neilsen, 1999; Milyaev *et al.*, 2022). Seed ablation in olive, which did not affect fruit development, also promoted back-flowering, supporting the above notion (Stutte and Martin, 1986; Lavee, 2007). Negative correlation between seed number per fruit and back-flowering intensity was also reported in citrus (Monselise and Goldschmidt, 1982; Davenport, 1990). However, in citrus, opposite trends have also been reported. Satsuma mandarin is seedless, yet it displays strong inhibition of back-flowering, and this is also the case in Ori mandarin, which has a very low number of seeds per fruit (Okuda, 2000; Schneider *et al.*, 2009). Furthermore, it should also be considered that dominance is exerted in parthenocarpic fruits. Therefore, it is likely that both the seed and the fruit provide the source of dominance and their relative contribution is species or even cultivar dependent.

## Other hormones

Besides auxin, other hormones play key roles in regulating shoot branching, particularly cytokinin and strigolactones. Cytokinin promotes branching, negating apical dominance, acting both to promote growth of the branch (Dun *et al.*, 2012) and to increase auxin sink strength in the stem (Waldie and Leyser, 2018). Conversely, strigolactones typically repress branching, enhancing apical dominance by inhibiting growth of the branch (Dun *et al.*, 2012) while decreasing sink strength in the stem (Crawford *et al.*, 2010; Shinohara *et al.*, 2013). It therefore seems very likely that other hormones will play a role in modulating fruit-driven feedback. However, in the case of strigolactones, there is little evidence either way. In Arabidopsis and *Brassica napus*, strigolactone-deficient mutants produce the same number of fruit and seed yield as wild-type, despite their strongly increased number of inflorescences (Walker and Bennett, 2018; Stanic *et al.* 2021), and undergo inflorescence arrest at the same time as wild-type (Ware *et al.*, 2020), suggesting that strigolactones might not be required for normal fruit-driven feedback, at least in the *Brassicaceae*. In other species, the effects of strigolactones on reproductive development have not been well studied, and the question thus remains open.

Conversely, there is some evidence for cytokinin acting to oppose fruit-driven feedback in the *Brassicaceae*. Cytokinin treatment of *Aethionema arabicum* increases the proportion of large fruit produced on each inflorescence (Lenser *et al.*, 2018), while cytokinin treatment of Arabidopsis delays inflorescence arrest (Merelo *et al.*, 2022; Walker *et al.*, 2022). In part this is because of the effect of cytokinin in prolonging the activity of the inflorescence meristem, but analysis of the *rock2* and *rock3* mutants with enhanced cytokinin signalling shows that cytokinin also promotes the opening of flowers (after inflorescence meristem arrest) that would normally be prevented by fruit-driven feedback (Ware *et al.*, 2020; Walker *et al.*, 2022). Cytokinin could also reverse first-fruit-inhibition phenomena in cucumber (Baniel *et al.*, 2008). Thus, in terms of mitigating the effects of fruit-driven feedback, enhanced cytokinin seems like an interesting route to explore to improve fruit crop yields.

Gibberellins and abscisic acid (ABA) might also play roles in controlling fruit-driven feedback, particularly in the complex effects of fruit load on flowering. Gibberellins, applied during the flowering induction period, are strong inhibitors of fruit tree flowering (Bangerth, 2009). The effect of external application of gibberellin on the expression of flowering-control genes is well established in several trees (Goldberg-Moeller *et al.*, 2013; Zhang *et al.*, 2019). It might well be that increased auxin levels in the floral buds of on-crop plants induce gibberellin synthesis (as is the case during fruit-set), and therefore flowering inhibition. In cucumber, application of GA could reverse the inhibitory effect of the first fruit on the development of later pollinated ovaries (Baniel *et al.*, 2008). ABA is also involved in flowering control, at least in citrus under stress conditions, and changes in ABA levels have been associated with fruit load in a few fruit tree species, including olive, citrus, and pistachio (Goldschmidt and Sadka 2021). However, the role of ABA in this process is currently unclear, and more investigation is required.

## Epigenetic mechanisms and fruit memory

An intriguing question is how fruit trees are able to ‘remember’ previous fruit loads, even when the fruit have been removed. Recent work in citrus suggests an explanation for this remarkable phenomenon. *CcMADS19* is a citrus orthologue of the floral repressor *FLOWERING LOCUS C* (*FLC*) from Arabidopsis, whose expression was shown to be reciprocal to that of the floral activator *FLOWERING LOCUS T* (*FT*) (Agustí *et al.*, 2020). In leaves of on-crop trees, transcript levels of *CcMADS19* are high, while those of *CcFT* are low, and vice versa in leaves of off-crop/de-fruited trees (Agustí *et al.*, 2020). Moreover, when transformed into citrus leaves, *CcMADS19* inhibited *FT* expression. Crucially, the methylation pattern of the *CcMADS19* large intron was found to be different in on-crop and off-crop trees, with de-fruited trees showing a similar pattern to off-crop trees (Agustí *et al.*, 2020). Use of a DNA methyltransferase inhibitor was sufficient to induce the expression of *CcMADS19* in on-crop trees, while reducing the expression of *FT*. Furthermore, on-crop trees were also found to have increased histone methylation (H3K4me3) in the *CcMADS19* promoter consistent with increased activity, and—consistent with this—to have increased expression of *TRITHORAX* proteins that act as *FLC* activators in Arabidopsis (Agustí *et al.*, 2020).

But how do the trees cycle between these epigenetic states, and what is the role of fruit in inducing this? Mesejo *et al.* (2022) suggest that differences in *CcMADS19* histone methylation between ‘old’ leaves and dormant axillary buds are crucial to this effect. In essence, ‘new’ tissues have H3K27me3 marks at the *CcMADS19* locus, making the gene inactive, whereas in older tissues the activating H3K4me3 mark predominates at this locus (Mesejo *et al.*, 2022). Removal of fruit, or just girdling of fruit peduncles in on-crop orange trees. results in reduced auxin export from fruit, and leads to sprouting of the dormant axillary buds adjacent to the fruit (Mesejo *et al.*, 2022). These axillary buds produce new leaves, and ultimately undergo flowering and give rise to new inflorescences. Because fruit auxin export inhibits the growth of nearby axillary buds, fruit therefore affect the overall balance of *CcMADS19* histone methylation across the tree, with fruit presence leading to a higher proportion of tissue with increased *CcMADS19* expression, and fruit absence leading to an increase in tissue with *CcMADS19* repression (Mesejo *et al.*, 2022). The growth of new tissues with *CcMADS19* repressed then provides a source of *CcFT* expression to induce flowering the following season, while the absence of new tissues and *CcFT* expression in on-crop trees leads to vegetative growth occurring the following season instead. These results provide a plausible scenario connecting fruit presence and auxin export to an epigenetic ‘memory’ of the fruit load in citrus, but it remains to be established whether these results are generalizable to other, more distantly related fruit trees.

## Measuring reproductive success

While the effects and mechanisms of fruit-driven feedback have been discussed above, an important question that we have skirted so far is why? Why do fruit exert this counter-intuitive inhibitory effect on the further production of fruit? The mostly likely answer is that this feedback allows plants a simple homeostatic mechanism by which to measure their own reproductive success, and to adjust their reproductive development to the circumstances. This reflects the inherent uncertainties that accompany flowering as a reproductive strategy, the main uncertainty being pollination, especially for insect-pollinated species. However, the potential losses of flowers and fruit to wind, rain, and herbivory also count as major uncertainties during reproduction. Amongst these uncertainties, plants must open enough flowers to guarantee a minimum reproductive success, without producing so many fruit that there are insufficient resources for them to all fully develop. The feedbacks described here provide a mechanism to steer a course between these goals. Consistent with overall reproductive strategies, two broad feedback strategies can be identified: over-flowering and under-flowering.

Over-flowering is most appropriate in plants where reproductive effort is largely predetermined, especially where there is a significant time gap between initiation of reproductive structures and actual fruit-set. Spring-blooming fruit trees provide the prime example of this strategy; inflorescences are initiated in autumn, and flower opening is a synchronous event in spring, with little ability to adjust reproductive effort at that point. To ensure sufficient fruit-set despite the uncertainty, there is little option but to ‘over-flower’, to open far more flowers than can ever give rise to successful fruit. Under such circumstances, there is no possibility to use fruit-set to adjust same-phase flowering (which began and ended months before), but there is a strong need for carpic dominance to allow selective shedding of excess fruit. Even with over-flowering and selective fruit-shed mechanisms in play, a wide range of reproductive success might result, and thus next-phase feedback allows plants to adjust their subsequent reproductive effort relative to the eventual success of the preceding reproductive cycle.

By contrast, under-flowering is most appropriate in plants with a continuous reproductive effort, in which events early in reproduction can influence later events. An under-flowerer’s initial effort produces just enough flowers to guarantee minimum seed set if there is ‘perfect’ pollination, thus avoiding unnecessary investment in excess flowers and fruit. However, if pollination is not perfect, a lack of feedback from early fruit will promote the stepwise production of additional inflorescences or flowers to fulfil the minimum requirement (Walker *et al.*, 2022). Since the plant can initiate approximately the correct number of flowers in the first instance, the need for fruit–fruit feedback is greatly reduced. Thus, Arabidopsis represents a clear example of an under-flowering strategy.

There are of course a whole range of possibilities between these two strategies, with *B. napus* representing a ‘mild over-flowering’ strategy. Flowering in *B. napus* is continual and adjustable, but seems to contain a large initial effort that produces somewhat too many flowers, if all are successfully pollinated (Tayo & Morgan, 1975). Carpic dominance is therefore present in *B. napus* to prevent too much fruit-set, but same-phase feedback is also present to allow the initiation of additional inflorescences if fruit-set is too low.

## Perspectives

Feedback between fruit and flowering provides important insights into the mechanisms by which plants flexibly and continually adjust their shoot growth to match environmental conditions. As discussed above, very similar dominance mechanisms play a key role in the regulation of vegetative shoot architecture. Indeed, an appealing possibility is that all shoot and reproductive architecture is ultimately regulated by a single, unified dominance mechanism, or at least a suite of fundamentally similar dominance mechanisms. Recent work in pear illustrates how auxin export from leaves is necessary to maintain the paradormancy of autumn-initiated inflorescences (Wei *et al*, 2022), indicating feedback from vegetative to reproductive organs, and demonstrating for the first time (to our knowledge) that leaves exert dominance over other tissues. Conversely, work in barley might indicate that the initiation of reproductive structures is able to suppress vegetative branching (tillering) in this crop (Zwirek *et al*, 2019).

**Fig. 6.**
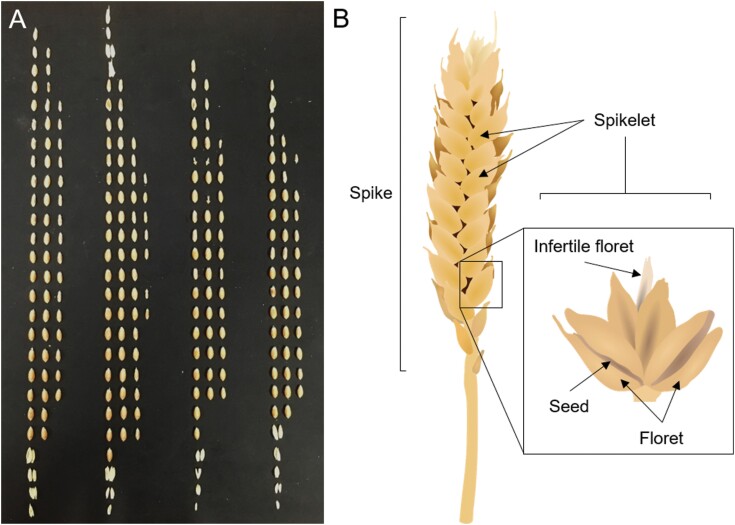
Possible dominance effects in wheat inflorescence development. The seeds from four ‘spikes’ from the same plants have been dissected out (A). Each row represents the seed from an individual spikelet (inflorescence) along the length of the spike (B). The medial spikelets develop first, and have a larger number of fertile flowers (i.e. seeds per spikelet), and larger seed than the apical and basal spikelets. Within each spikelet, the earlier flowers (on the left of each row) from the outer florets of the spikelet (see diagram, right) have larger seed than later-opening flowers.

More generally, cereals crops represent an interesting opportunity in terms of fruit-driven feedback. There is no doubting the importance of fruit-driven feedback in commercial fruit trees and horticultural crops, but what about arable crops? Do they also display such feedback, and to what extent could applying the frameworks outlined here to cereals revolutionize our understanding of their yield formation? Bangerth (1989) certainly thought that dominance relationships occur within the ‘spikes’ (grouped inflorescences) of cereals, but despite much interest in understanding the characteristic development and shape of cereal spikes (Wang *et al.*, 2021), there has been little subsequent work on dominance effects in cereals. We can speculate the cereals are over-flowerers, on the basis that the initiation and complete development of spikes occurs a significant time before the spikes emerge into the air, and undergo anthesis and pollination. There is therefore little scope for cereals to produce more flowers except by ‘back-tillering’ during flowering and initiating new, later spikes. Consistent with this idea, we can see that, within a wheat spike, there are strong gradients of grain size (the grains are a caryopsis, a fruit containing a single seed in which the fruit and seed tissues are fused together). This occurs both within spikelets (the small inflorescences that make up the spike), which could indicate older grain inhibiting the growth of younger grain, and between spikelets, which could be indicative of older spikelets inhibiting the growth of later ones (Fig. 6). This would be broadly consistent with an over-flowering strategy. Clearly, more work is needed to explore fruit-driven feedback in the context of cereals; if found to be relevant, there is certainly huge scope to improve grain size across a typical cereal spike.

Mitigating fruit-flower feedback inhibition has long been a subject of intensive research in fruit trees. Numerous approaches have been developed to try and improve fruit tree yields, mostly based on plant growth regulators or alternation of sink–source relations (reviewed in Goldschmidt and Sadka, 2021). For instance, fruitlet thinning during the on-crop year using synthetic auxin compounds is well established in many fruit trees. Manipulating hormonal content during flowering induction period and during flowering time and fruit set is also well documented. Girdling, which induces sugar content in the canopy, might well mitigate previous fruit effects on flowering, and pruning is also a common practice. In line with the notion that auxin canalization is the origin of dominance, use of inhibitors of polar auxin transport, such as tri-iodobenzoic acid, has also been shown to mitigate fruit–flower feedback inhibition (Fichtner *et al.*, 2021). However, deeper understanding of the underlying molecular mechanisms will hopefully in the future allow more stable, genetic manipulation of fruit-tree germplasm, in order to overcome the self-limiting effects controlling fruit–flower feedback without labour-intensive practices.

## Data Availability

There are no primary data associated with this manuscript.
